# Research Data Management and Data Sharing for Reproducible Research—Results of a Community Survey of the German National Research Data Infrastructure Initiative Neuroscience

**DOI:** 10.1523/ENEURO.0215-22.2023

**Published:** 2023-02-13

**Authors:** Carsten M. Klingner, Michael Denker, Sonja Grün, Michael Hanke, Steffen Oeltze-Jafra, Frank W. Ohl, Janina Radny, Stefan Rotter, Hansjörg Scherberger, Alexandra Stein, Thomas Wachtler, Otto W. Witte, Petra Ritter

**Affiliations:** 1 Hans Berger Department of Neurology, Jena University Hospital, Jena, 07747, Germany; 2 Biomagnetic Center, Jena University Hospital, Jena, 07747, Germany; 3 Berlin Institute of Health at Charité - Universitätsmedizin Berlin, Berlin, 10117, Germany; 4 Department of Neurology with Experimental Neurology, Brain Simulation Section, Charité - Universitätsmedizin Berlin, Corporate Member of Freie Universität Berlin and Humboldt-Universität zu Berlin, Berlin, 10117, Germany; 5 Bernstein Center for Computational Neuroscience Berlin, Berlin, 10117, Germany; 6 Einstein Center for Neuroscience Berlin, Berlin, 10117, Germany; 7 Einstein Center Digital Future, Berlin, 10117, Germany; 8 Bernstein Coordination Site, Jülich, 79104, Germany; 9 University of Freiburg, Freiburg im Breisgau, 79098, Germany; 10 Faculty of Biology, Ludwig-Maximilians-Universität München, München, 82152, Germany; 11 Deutsches Primatenzentrum GmbH – Leibniz-Institut für Primatenforschung, Göttingen, 37077, Germany; 12 Faculty of Biology and Psychology, University of Göttingen, Göttingen, 37073, Germany; 13 Institute of Neuroscience and Medicine, Brain & Behaviour (INM-7), Research Center Jülich, Jülich, 52428, Germany; 14 Institute of Systems Neuroscience, Medical Faculty, Heinrich Heine University Düsseldorf, Düsseldorf, 40225, Germany; 15 Bernstein Center Freiburg and Faculty of Biology, University of Freiburg, Freiburg im Breisgau, 79104, Germany; 16 Institute of Neuroscience and Medicine (INM-6) and Institute for Advanced Simulation (IAS-6) and JARA-Institute Brain Structure-Function Relationships (INM-10), Jülich Research Centre, Jülich, 52428, Germany; 17 Theoretical Systems Neurobiology, RWTH Aachen University, Aachen, 52074, Germany; 18 Department of Neurology, Otto von Guericke University, Magdeburg, 39120, Germany; 19 Peter L. Reichertz Institute for Medical Informatics, Hannover Medical School, Hannover, 30625, Germany; 20 Leibniz Institute for Neurobiology (LIN), Magdeburg, 38118, Germany; 21 Center for Behavioral Brain Science (CBBS), Magdeburg, 39106, Germany

**Keywords:** research data infrastructure, data sharing, metadata, survey, community, information security and privacy

## Abstract

Science is changing: the volume and complexity of data are increasing, the number of studies is growing and the goal of achieving reproducible results requires new solutions for scientific data management. In the field of neuroscience, the German National Research Data Infrastructure (NFDI-Neuro) initiative aims to develop sustainable solutions for research data management (RDM). To obtain an understanding of the present RDM situation in the neuroscience community, NFDI-Neuro conducted a comprehensive survey among the neuroscience community. Here, we report and analyze the results of the survey. We focused the survey and our analysis on current needs, challenges, and opinions about RDM. The German neuroscience community perceives barriers with respect to RDM and data sharing mainly linked to (1) lack of data and metadata standards, (2) lack of community adopted provenance tracking methods, (3) lack of secure and privacy preserving research infrastructure for sensitive data, (4) lack of RDM literacy, and (5) lack of resources (time, personnel, money) for proper RDM. However, an overwhelming majority of community members (91%) indicated that they would be willing to share their data with other researchers and are interested to increase their RDM skills. Taking advantage of this willingness and overcoming the existing barriers requires the systematic development of standards, tools, and infrastructure, the provision of training, education, and support, as well as additional resources for RDM to the research community and a constant dialogue with relevant stakeholders including policy makers to leverage of a culture change through adapted incentivization and regulation.

## Significance Statement

A comprehensive survey among the neuroscience community in Germany determined the current needs, challenges, and opinions with respect to standardized research data management (RDM). The Neuroscience community perceives a lack of standards for data and metadata, a lack of provenance tracking and versioning of data, a lack of protected digital research infrastructure for sensitive data and a lack of education and resources for proper RDM. However, an overwhelming majority of community members indicated that they would be willing to share their data with other researchers and are interested to increase their RDM skills. Thus, the survey results suggest that training, the provision of standards, tools, infrastructure, and resources for RDM holds the potential to significantly facilitate reproducible research in neuroscience.

## Introduction

Annual brain health costs exceed €800 billion in Europe (DiLuca and Olesen, 2014). Many factors contribute to the difficulty of developing effective treatments for brain diseases. These include the gaps in knowledge about the precise changes and biological processes in the brain that cause a disease, and the long time needed to observe whether an investigational treatment affects disease progression. Many studies have been collecting cohort datasets in patients with brain disease to better understand the mechanistic basis of the disease, qualify diagnostic and monitoring biomarkers, and test drugs. Given that most studies are typically limited in their range of assessments, there is tremendous value in combining and integrating the resulting data (Milham et al., 2018). Multimodal data across different studies can further been used to construct integrated *in silico* models of the brain and multiscale potential targets for multilevel interventions. However, the proportion of scientific data that is actually openly shared within the neuroscientific community remains low (Watson, 2022). The lack of sharing properly annotated data and tools contributes to the poor reproducibility of research results, known as “the reproducibility crisis,” that hinders the growth of knowledge and innovation on the one hand and leads to inefficient use of resources on the other hand (Baker, 2016; Stodden et al., 2016; Poldrack et al., 2019; Crook et al., 2020; Loss et al., 2021; Niso et al., 2022).

The German National Research Data Infrastructure Initiative (NFDI) implemented by the German Research Foundation (DFG) will provide up to €85 million per year over the course of 10 years (https://dfg.de/nfdi) to foster research data management (RDM) across all research domains in Germany. RDM describes the organization, storage, preservation, and sharing of scientific data. This includes the day-to-day management of research data during the lifetime of a research project and the long-term usability of these data through the FAIR principles (findable, accessible, interoperable, and reusable). NFDI comprises domain-specific consortia across all science disciplines. In the field of neuroscience, the initiative NFDI Neuroscience (NFDI-Neuro; https://nfdi-neuro.de) initiative has started to closely interact with the neuroscience community to overcome the challenges in RDM (Denker et al., 2021a,b; Hanke et al., 2021; Klingner et al., 2021; Wachtler et al., 2021).

The NFDI-Neuro initiative is aligned to several international programs such as the WHO Global strategy on digital health, the European Health Data Space, the European Interoperability Framework (EIF) and the Digital Europe Program (https://digital-strategy.ec.europa.eu/en/activities/digital-programme) by addressing topics such as interoperability, fair digital objects, artificial intelligence, and cybersecurity. A goal of NFDI-Neuro is to foster the reproducibility of research and to leverage computational neuroscience as data integrating discipline that transforms data into knowledge and understanding.

To obtain a comprehensive understanding of the present RDM situation in the neuroscience community, NFDI-Neuro conducted a community survey to investigate what among neuroscience community is perceived as the largest obstacles and most pressing needs with respect to RDM, and how members of the community self-assess their present proficiency in RDM topics.

## Materials and Methods

The NFDI-Neuro community survey was developed based on a previous survey by the partner consortium NFDI4Bioimage (https://nfdi4bioimage.de/). It was adapted by the NFDI-Neuro team to address questions specific to the neuroscience research domain. The NFDI-Neuro survey comprised 20 sets of questions, where each set contained one or multiple questions. Counting all questions yields a total of 114 questions presented to each survey participant. The time required for answering all questions was 10–15 min. We used the tool LimeSurvey (https://www.limesurvey.org/de/) and conducted the survey online in compliance with the EU General Data Protection Regulations (GDPR). The online questionnaire was made available for two months between September 1, 2021 and November 1, 2021 via the website of the NFDI-Neuro initiative (https://nfdi-neuro.de/). We announced the survey via several channels, including E-mail lists of the German neuroscience communities, such as the German Society for Clinical Neurophysiology, the German Neuroscience Society, and the Bernstein Network Computational Neuroscience, as well as the NFDI-Neuro community mailing list and Twitter channel (https://twitter.com/NFDI_Neuro). In total, 218 individuals of either sex participated in the online survey. Of those, 85 participants did not answer all questions. We included in our analysis all given answers, including those of the incomplete questionnaires. For the data analysis and the generation of the figures, we used the software package R [version 4.1.2 (“Bird Hippie”)]. The survey and related collected data, as well as all analysis scripts are available publicly (https://doi.org/10.12751/g-node.w5h68v).

### Data availability

Raw data of the survey have been published in a research data repository (https://doi.org/10.12751/g-node.w5h68v).

## Results

In the following, we present the main results of the survey. A visual representation of the answers to each question can be found in the supplementary material.

### Participants represent a broad range of neuroscience disciplines

**Figure 1.**
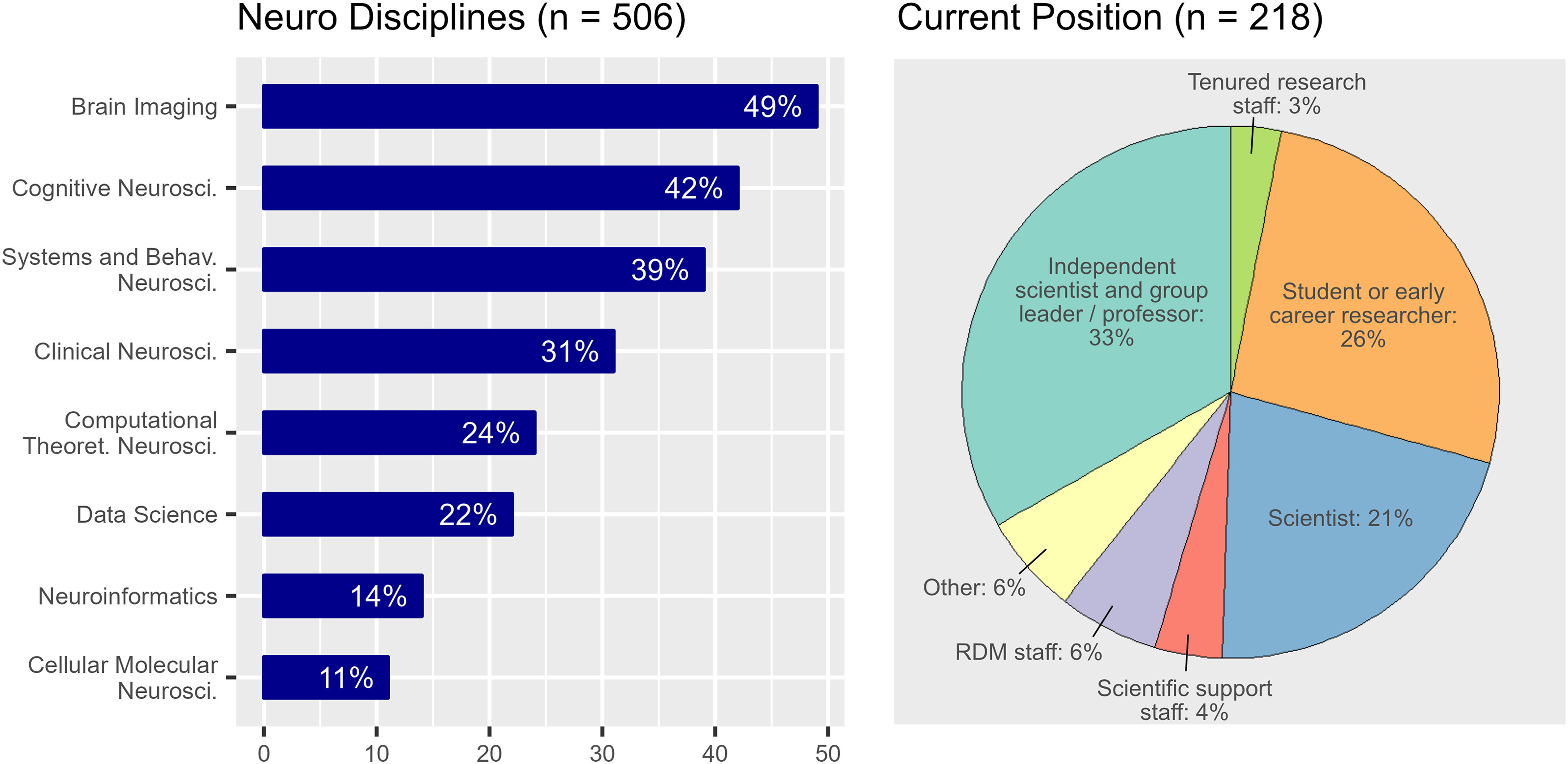
Distribution of neuroscience subdisciplines (multiple answers allowed, left), professional position of participants (right).

Most respondents work at a public university or government research institution (71%), while 19% work at a nonprofit research institute and 5% work at a private company. The distribution of professional positions of the survey participants shows a tendency toward higher positions in the scientific hierarchy, with 73 (33%) “Independent scientist and group leader/professor,” 46 (21%) “Scientists,” 56 (26%) “Student or early career researcher,” 14 (6%) “Research data management focused staff,” 6 (3%) “Tenured research staff,” 9 (4%) “Scientific support staff,” 14 (6%) “Other” (Fig. 1). The participants cover a wide range of neuroscience subdisciplines (Fig. 1; selection of multiple choices possible) led by brain imaging (106, 49%) followed by cognitive neuroscience (92, 42%), systems and behavioral neuroscience (84, 39%), clinical neuroscience (67, 31%), computational/theoretical neuroscience (53, 24%), data science (48, 22%), neuroinformatics (31, 14%), and cellular/molecular neuroscience (25, 11%).

### A considerable amount of research data is not yet being shared

**Figure 2.**
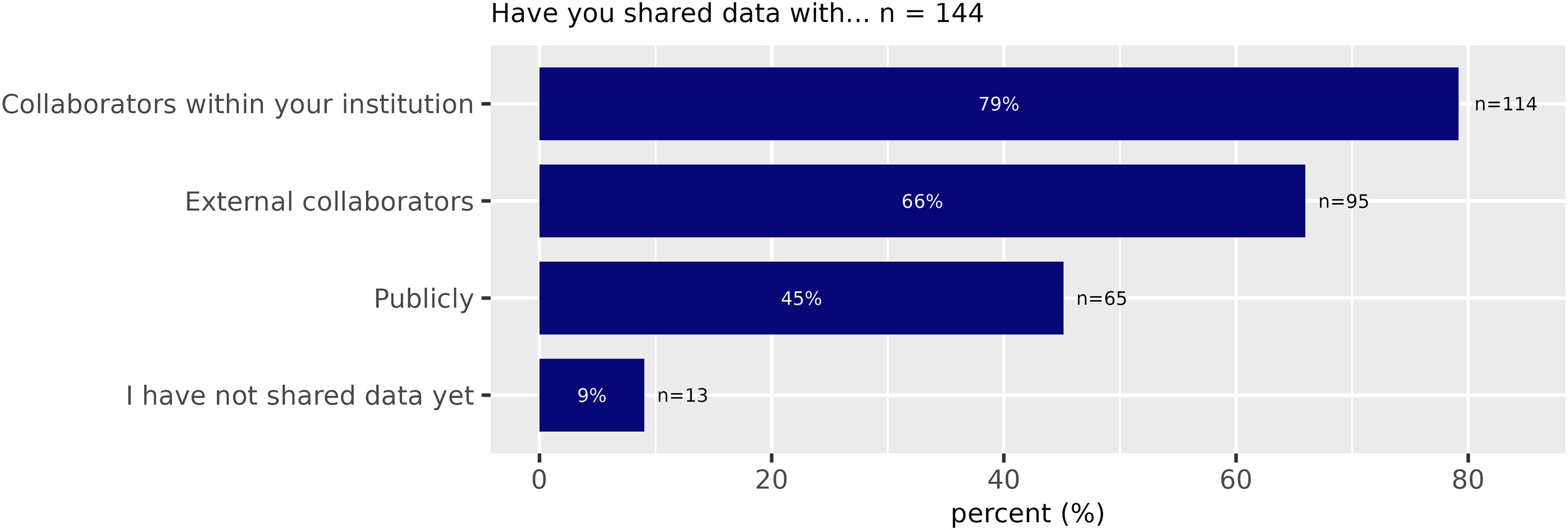
Data sharing (Survey Question 7).

**Figure 3.**
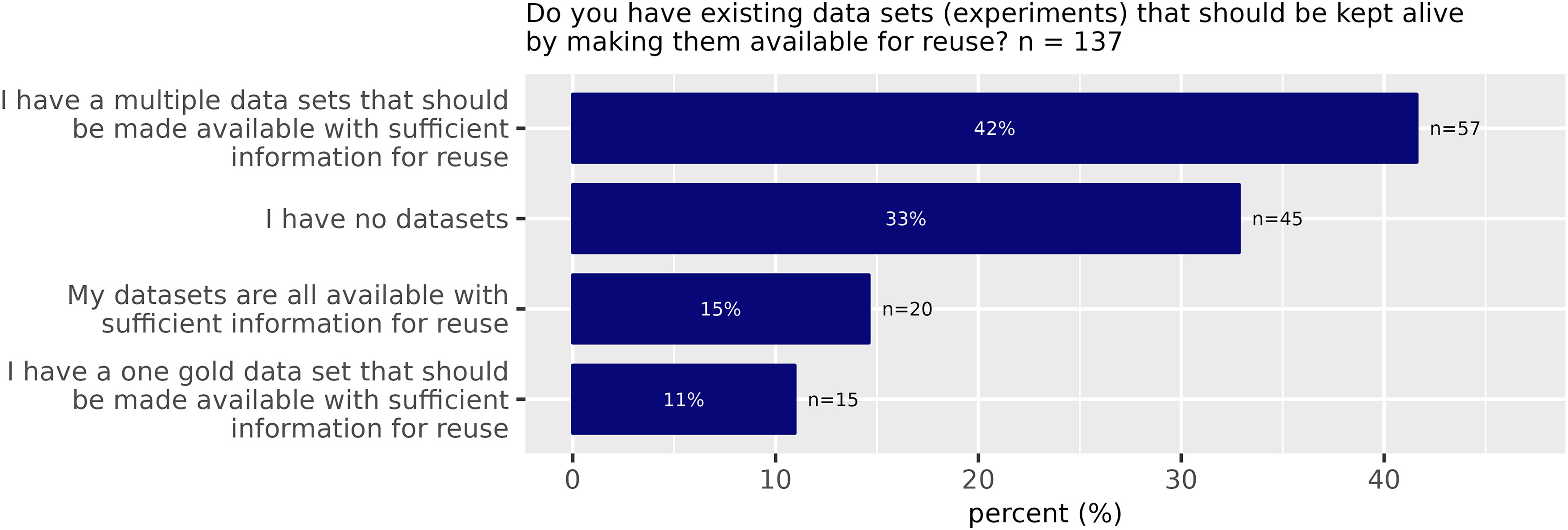
Existing datasets (Survey Question 8).

A total of 114 (79%) of all participants indicated that they share data within their institution. 95 (66%) share their data with external collaborators while only 65 (45%) share data publicly, that is they made datasets openly discoverable via repositories (at least one dataset). Only 13 participants (9%) had never shared any data yet (Fig. 2). A primary objective of the NFDI initiative is to improve the reuse of research data. In this context, we explored the potential availability of neuroscience data that is not yet shared publicly but is considered of general interest. We asked whether the participants own data of potential interest to other scientists for reuse (Fig. 3). According to the responses, 84 (67%) of the participants have valuable datasets available that would be useful for further exploitation, but only 20 (22%) of those participants make these data available for reuse. 76 (84%) of all participants with at least one dataset believed that other researchers could answer their research questions by reusing data from their research. However, even for this subgroup of scientists that think their data are valuable to others, 48% have never publicly (via public repositories) shared any of their data.

### Own data management skills are commonly seen as not being high

**Figure 4.**
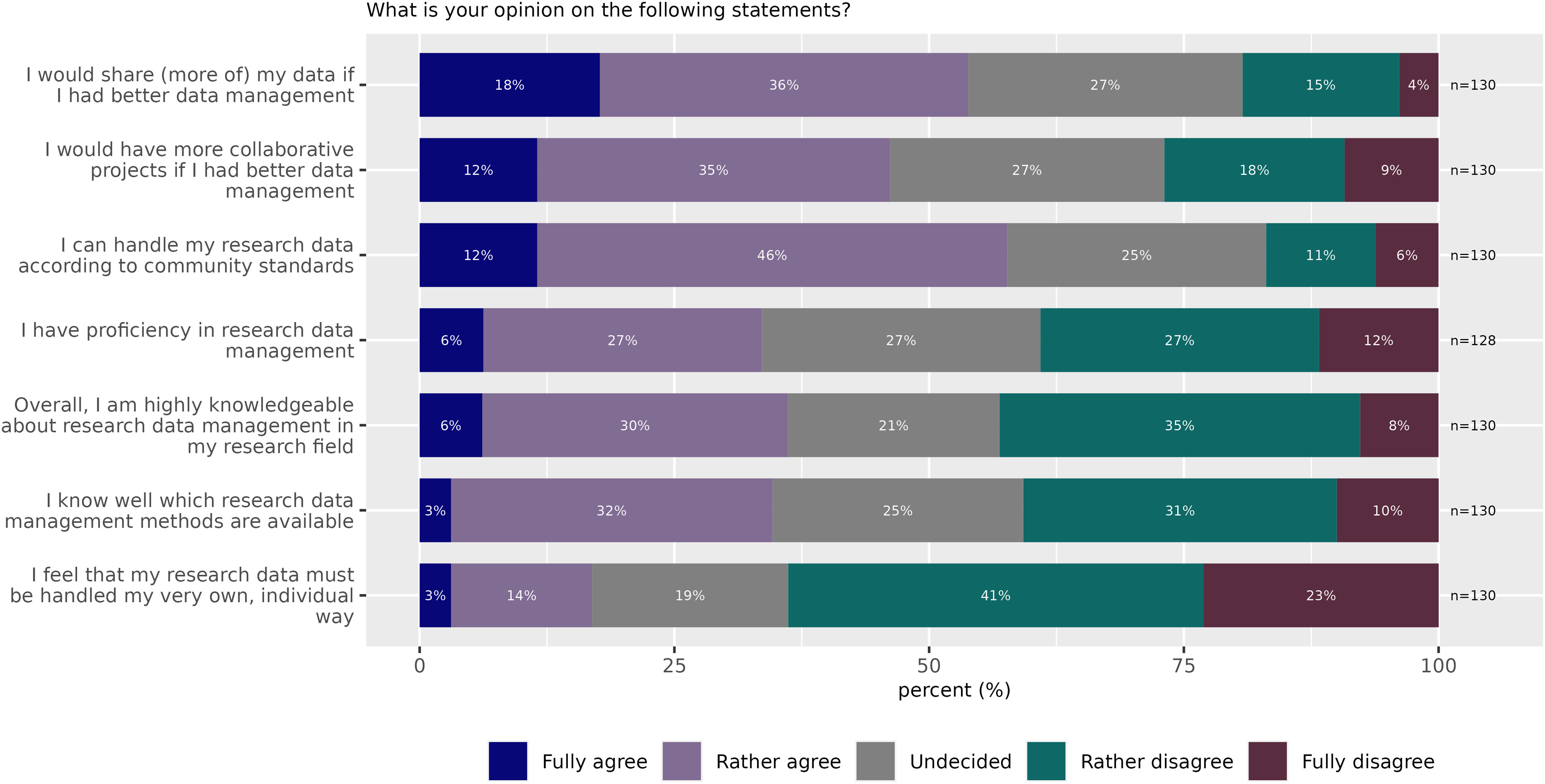
Perceived obstacles for RDM and data sharing (Survey Question 14).

Research data management skills are essential for preparing, analyzing, and publicly sharing data. 43% of responders disagreed with the statement “Overall I am highly knowledgeable about research data management in my research field” (Fig. 4). Only 33% of the survey participants thought that they have proficiency in research data management. Only 36% thought they know which research data management methods are available, and 36% thought they are “highly knowledgeable about research data management.” Interestingly, 58% of all respondents nevertheless agreed or rather agreed that they “can handle their research data according to community standards.” It is unlikely that this apparent discrepancy could be explained by the availability of data research managers who assist with data handling because only 19% of participants indicated that they have dedicated personnel with research data management or data curation expertise in their labs.

We further investigated whether there is a dependency between public data sharing and the self-perception of competence regarding RDM. From the reported self-assessments, only the statement “I think that I can handle research data according to community standards” (Fig. 4) showed a strong connection to the response that data are shared openly (Fig. 2). Participants agreeing to this statement were six times more likely to share data publicly than those who were disagreeing. Self-assessed high competence in the other RDM capabilities correlated with reported data sharing as well, but to lower degrees (“I know which research data management methods are available” 1.2-fold, “Overall, I am highly knowledgeable about research data management in my research field” 1.4-fold, “I have proficiency in RDM” 1.75-fold). Thus, in summary, the higher the level of RDM knowledge, the higher the level of data sharing.

### Tools and standards for RDM are not yet widely used

**Figure 5.**
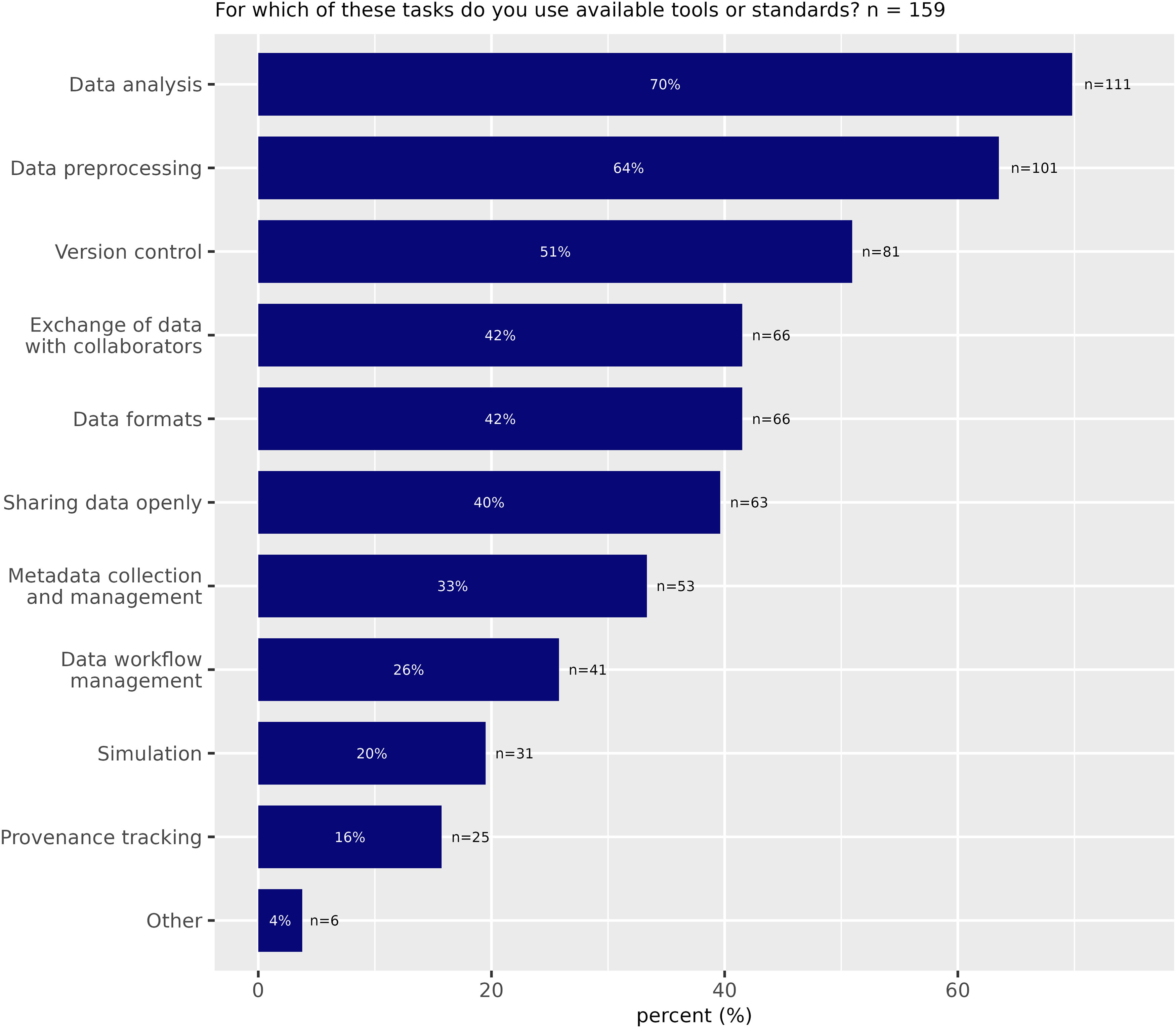
Use of existing tools and standards for different research data management activities (Survey Question 5).

While the responses indicated that standard tools for data processing and analysis are widely used, the use of standard RDM tools for data sharing was reported to significantly lower extent (sharing data openly, metadata collection and management, provenance tracking; Fig. 5).

### Scientists who use more tools or standards are more likely to share their data

In the group that indicated not sharing their data publicly, only 33% indicated using tools or standards, while this proportion was 54% in the group of data sharers. A possible explanation could be that scientists who work a lot with standard tools find it easier to present their data in a shareable form and have a higher digital literacy required for the public sharing of data. Alternatively, the motivation to share data may be a strong driver to adopt standard methods. Respondents who indicated sharing their data publicly were 42% more likely to use standard tools “mostly” in their daily work compared with respondents who did not share their data publicly.

### Perceived obstacles for research data management and sharing

Reluctancy to share data publicly because the data ownership or intellectual property might be violated was indicated by 20% of respondents. Interestingly, 37% of participants reported that they do not know whether their institutional policy allows uploading data to a public repository, while only 9% were confident that their institutions do not support this.

Further, 58% were not sure whether they own the rights to upload data from their own research project; 48% reported seeing legal aspects as significant hurdles for public repository usage. These answers indicate that substantial uncertainties about legal issues regarding data sharing exist in the research community. This is confirmed by only 18% indicating that legal aspects were not perceived as significant hurdles for public repository usage.

Only 29% of participants thought there is sufficient guidance for choosing an appropriate repository for their data; 63% believed that there is a lack of expertise and human resources to deposit data in a repository; 45% thought that the technical hurdles are too high to upload data to a repository.

Eighty-three percent of respondents indicated that their research data did not have to be handled in an individual way not easily compatible with existing standards, tools, or guidelines (Fig. 4). The lack of professional data management was reported as a problem. A total of 70 (54%) participants responded that they would share more of their data if they had better RDM, while only 27% indicated that better RDM would not increase the amount of their own data to share.

**Figure 6.**
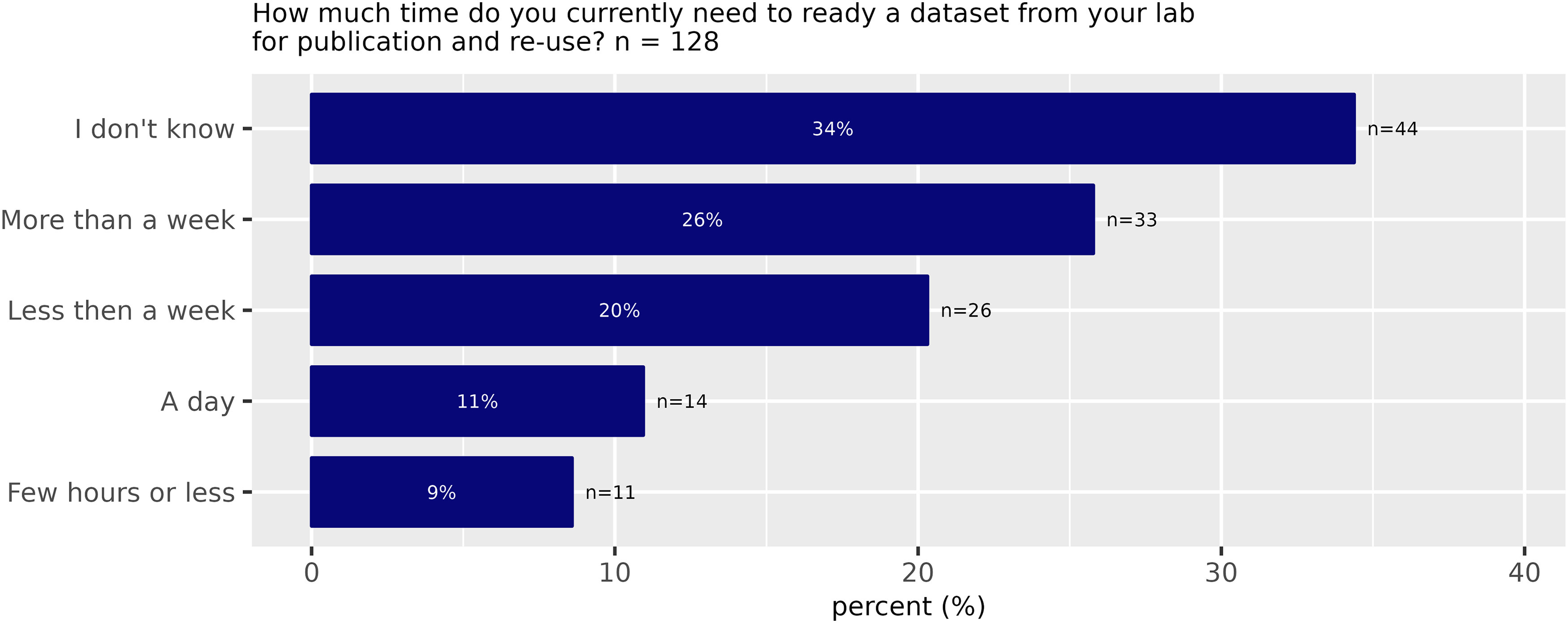
Required time to prepare a dataset for publication and reuse (Survey Question 18).

A total of 70% of those respondents who had previously prepared data for publication and reuse indicated that the time they need to ready a dataset requires more than a day, and 26% need even more than a week (Fig. 6).

Accordingly, 60% thought that there is lack of time to deposit data in a repository. In comparison, only 23% did not believe that time is a problem for depositing data in a public repository.

Questioned for the most pressing issues hindering research data management and public data sharing, there was a strong consensus. Close to 70% of respondents rated the following problems as one of the top three:

- “Inappropriately documented custom code in non-reproducible computational environments”
- “Poor standardization of metadata and derived data”

Other concerns were indicated similarly often but were rated with lower importance compared with the top two problems:

- “Lack of automatic data quality control”
- “Harmonization and fusion of data from multiple sites and/or studies”
- “No standardized support for concerted study data and metadata extraction from multiple devices and data linking”
- “Data security issues in data exchange with other institution”

In addition, the responses suggested that general knowledge about methods and tools of research data management is lacking. Only 34% of participants indicate that they think they know which RDM methods are available.

### Factors promoting public data sharing

**Figure 7.**
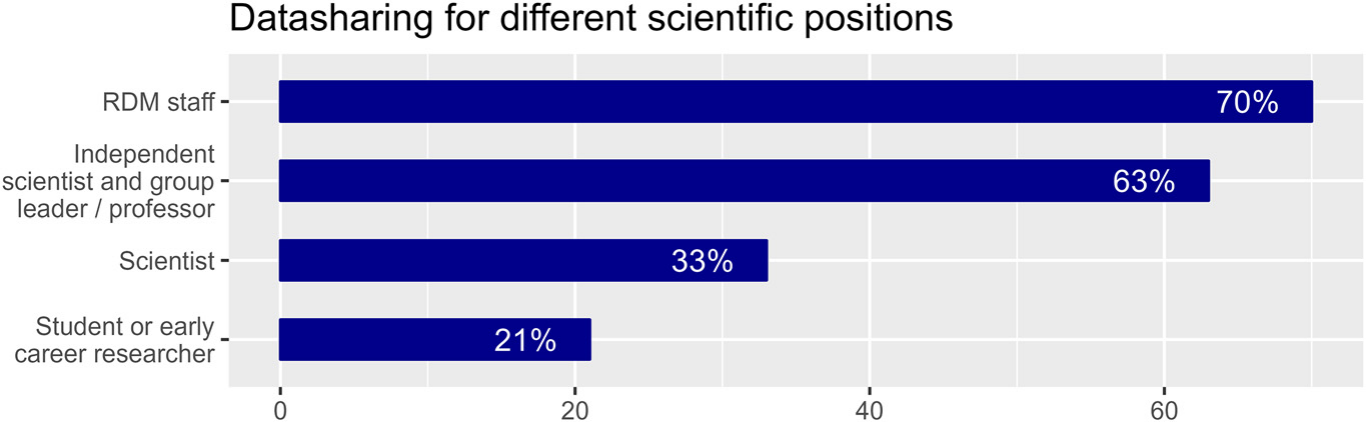
Percentage of respondents that have at least one dataset shared publicly shown separately according to their scientific position.

To identify factors that promote public data sharing, we analyzed separately the answers of the participants who had already shared their data in public repositories (*n* = 65). For this analysis we excluded responses from participants who had indicated that they had never shared a single dataset publicly. The proportions of the different academic groups among scientists who have shared data publicly varied considerably: responses to the question whether data are shared depended on the position and experience of the person managing the data (Fig. 7).

Interestingly, whether dedicated personnel with RDM or data curation expertise is available seems not to affect whether data are publicly shared. Public data sharing was reported by 56% of those indicating that dedicated RDM personnel is available and by 54% of those indicating that it was not available.

**Figure 8.**
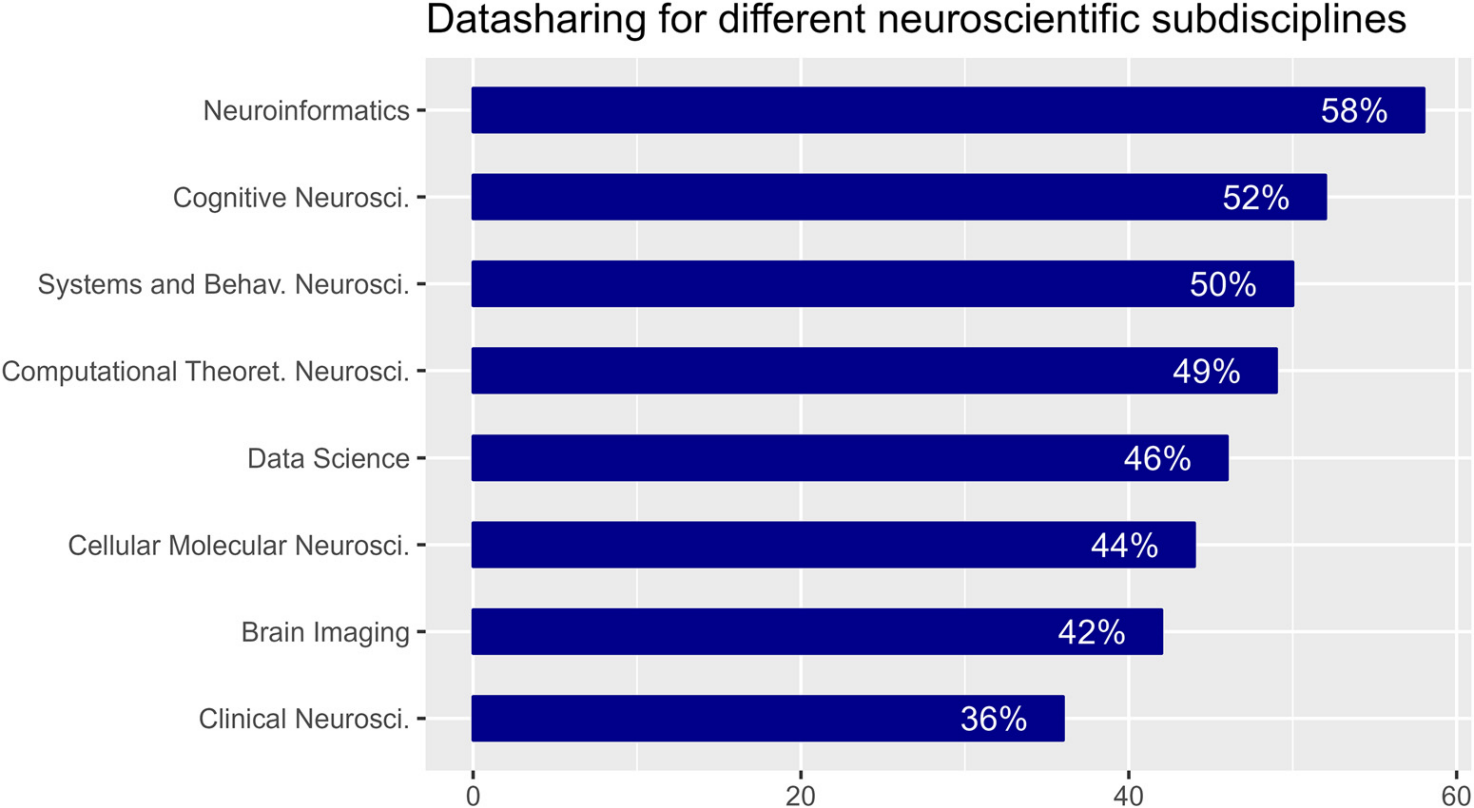
Percentage of respondents who have at least one dataset shared publicly, separated according to their scientific subdomain.

We analyzed the dependence between the willingness to share data and the scientific subdomain of the respective researcher. We found a relatively high degree of data sharing indicated by scientists in the subdomain of neuroinformatics (58%), and a relatively low degree (36%) of data sharing in clinical neuroscience (Fig. 8).

## Discussion

It is generally recommended and expected by journals and founding agencies that scientific data be shared to improve collaboration, transparency, and reproducibility in science. However, less than half (45%) of the participants of the current survey stated they had made at least one dataset publicly available. While this suggests that data sharing is possible in principle, it remains unclear to what extent this 45% of scientists share the data they collect. In any case, most participants have never shared data publicly. Although the survey was conducted anonymously, there is nevertheless a tendency to answer according to social desirability or even to give an answer bias in the direction that the respondent himself would like to see. Therefore, the proportion of scientists who did not share data could be even higher. In addition, it remains unclear how much of the data they collected they shared, as a single publicly shared dataset is enough to be counted in this group. The percentage of collected data that is publicly shared remains therefore unclear but is certainly well below 45%. This result is consistent with a previous survey on open science practices in functional neuroimaging which reported that 34% of their participants have never shared their raw neuroimaging data (Paret et al., 2021). This similarity could also be caused by the fact that half of respondents in our survey are engaged in neuroimaging. Some other domains might be underrepresented like cellular molecular neuroscience (11%) or neuroinformatics (14%). The sample size of the survey is comparable to other surveys in this area and similarity of results indicate representativeness (Niso et al., 2022). The low rate of shared data can be explained by the fact that scientists do not want to or cannot share the data or that there are at least barriers that ultimately lead to the data not being shared. Respondents to our survey showed no fundamental objection to sharing and reusing scientific data. Yet the data-collecting scientist may fear specific disadvantages from sharing data, e.g., other scientists can specialize in refuting study results and make their mark at the expense of the scientists collecting the data (Longo and Drazen, 2016). Other authors argue that sharing data are worthwhile even for the “most avaricious and self-interested scientist” and leads to an improvement in their own scientific productivity and career advancement (Hunt, 2019).

Even if there is no fundamental resistance to sharing data, the results indicate that there are barriers to sharing data. In the survey, we specifically addressed respondent’s perceptions of various possible barriers. Reluctancy to share data publicly because the data ownership or intellectual property might be violated was indicated by 20% of respondents. Interestingly, more than one-third of participants reported that they do not know whether their institutional policy allows uploading data to a public repository, while only one out of ten was confident that his/her institution do not support this.

Further, more than half of all respondents were not sure whether they own the rights to upload data from their own research project. A total of 48% reported seeing legal aspects as significant hurdles for public repository usage. These answers indicate that substantial uncertainties about legal issues regarding data sharing exist in the research community. This is confirmed by only a minority (18%) state that legal aspects were not perceived as significant hurdles for public repository usage. This general view is consistent with a recent review of the spectrum of data sharing policies in neuroimaging data repositories (Jwa and Poldrack, 2022). The authors highlighted the complexity of ethical and legal issues related to neuroimaging data in the United States, which depend on several factors, such as the sensitivity of the data, whether a federal agency is involved, or whether stakeholders still want to retain some control over the shared data. Because of the complexity of the solutions to the constraints and requirements, choosing the right solution requires expert knowledge, and the increasing threats posed by new technologies to the privacy of shared data lead the authors to propose a legal ban on the malicious use of neuroscience data (Jwa and Poldrack, 2022). In line with this complexity, only 29% of participants thought there is sufficient guidance for choosing an appropriate repository for their data; 63% believed that there is a lack of expertise and human resources to deposit data in a repository. However, beyond legal and ethical issues, 45% thought that also the technical hurdles are too high to upload data to a repository.

These answers do not indicate the lack of a specific solution that is urgently needed, but rather that the problem lies in the difficulty of finding and applying the right solution for the individual case.

### Insufficient incentives to spend the time needed for RDM

Barriers to sharing data appear also in the requirement of data preparation, which can be a time-consuming process. A majority (70%) of those respondents who had previously prepared data for publication and reuse indicated that the time they need to ready a dataset requires more than a day, and 26% need even more than a week (Fig. 6). Accordingly, 60% thought that there is lack of time to deposit data in a repository. In comparison, only a quarter (23%) did not believe that time is a problem for depositing data in a public repository. In other words, nearly half (40%) of scientists state it takes them less than a week to prepare a dataset for publication and reuse. The fact that the majority (60%) nevertheless think there is a lack of time suggests that scientists do not think the time investment is worth it. The lack of time results from competing demands, each of which requires time. Given that scientists do science, this result suggests that preparing data for publication and reuse competes with the act of doing science, which itself is not always perceived as a central part of science.

In any case, however, the reports of lack of time indicate that scientists perceive other tasks as more important than data sharing. Accordingly, this reflects an insufficient incentive to spend the time needed to prepare data for publication and reuse. On the one hand, the incentive can be increased but also the time required can be reduced to make the ratio of data sharing more advantageous. The time required can be reduced through the use of tools and the competence in research data management can be increased.

### Need for training and education

The fact that two-thirds of the participants do not consider themselves competent in this area and think they do not know which research data management methods are available shows that there is considerable potential for optimization here. Training in this regard is particularly important as our analysis demonstrated a strong correlation between research data management skills and the amount of data sharing. Research data management skills also saving time which is perceived as a major barrier to data sharing. Training and education should also convey the value of data sharing to the scientists themselves. In a recent Nature RDM survey (Checklists Work to Improve Science, 2018), 58% of participants think that the researchers hold the key role for improving the reproducibility of research and 91% see them among the top three stakeholders to achieve this – thus being in a leading role ahead of laboratory heads, publishers, funders, department heads and professional societies who also were among the choices. This is in great alignment with what we experience in our work within RDM neuroscience projects [NFDI-Neuro, INCF, EBRAINS, central informatics projects of collaborative research centers funded by DFG (known as “INF projects”): “It is the researchers themselves who are required to do the RDM and to co-develop RDM tools - and hence require training to obtain RDM literacy.” “Reproducibility is like brushing your teeth. Once you learnt it, it becomes a habit” (Irakli Loladze in Baker, 2016)]. NFDI-Neuro aims to bring RDM to the individual labs, via several mechanisms including the establishment of transfer teams, working groups and massive training offers.

### Summary of perceived needs based on the survey results

The results of this survey indicate the following five task areas for improving RDM in Neuroscience:

1. Training, education, and networking activities.
2. Data and metadata standards, to advance and disseminate existing standards.
3. Provenance and workflows to advance and disseminate solutions for data lineage and digital reproducible workflows.
4. Infrastructure and service, for data management and processing, including for sensitive data with Cloud and HPC resources.
5. Modelling and big data analytics, for collecting RDM requirements from the perspective of the secondary data user community.

### Limitation

Participants in this survey were not given any further information about the meaning of each term. The survey was answered as the participants understood the terms. Different levels of knowledge and also different subdisciplines could have an influence on the understanding of the terminology which in turn could affect the interpretation of the results. The subdisciplines of neuroscience were not evenly distributed among the respondents. The results of this survey can therefore not be generalized to all subdisciplines. The positions of the respondents showed a tendency toward more senior staff participating in the survey compared with the assumed distribution of positions in neuroscience research.

### Conclusion

With the present survey, we identified various challenges in RDM in the neuroscience community. We found that the community perceives significant deficits with respect to transparent and reproducible data handling, annotation and sharing. According to the survey results, researchers with more experience and knowledge in RDM are more likely to share data for secondary use by their colleagues.

In summary:

- Only one-third of neuroscientists think they have proficiency in RDM.
- Less than a quarter of the research teams have RDM staff.
- More than a third do not know whether institutional policies allow loading data to a repository.
- Two-thirds are not sure they own rights for uploading data to public repositories.
- Half of the researchers see legal hurdles for data sharing.
- Forty percent of those researchers who have previously prepared data for publication and reuse say that the time they need to ready a dataset requires more than a week.
- Sixty percent think there is a lack of time to deposit data in a repository.
- Only one-third think they know which RDM methods are available.

We are encouraged by the fact that only a minority of one-fifth of respondents in the neuroscience community are not inclined to share data for reuse and that literacy in the usage of tools and standards increases the frequency of data sharing. Thus, the survey results suggest that training, the provision of properly secure and protected research infrastructure, tools, standards, and additional resources for RDM are promising approaches to leverage RDM and foster reproducible and efficient research practices in neuroscience. NFDI-Neuro will deliver on these topics. Therefore, we are convinced that we are addressing with NFDI Neuro the most pressing needs of our community. Our initiative has contributed significantly to several of the crosscutting goals of NFDI in the past. NFDI-Neuro plans to advance these operational solutions and to transfer them to an increasing number of labs of the German and international science community.

## Synthesis

Reviewing Editor: William Stacey, University of Michigan Decisions are customarily a result of the Reviewing Editor and the peer reviewers coming together and discussing their recommendations until a consensus is reached. When revisions are invited, a fact-based synthesis statement explaining their decision and outlining what is needed to prepare a revision will be listed below. The following reviewer(s) agreed to reveal their identity: Oscar Esteban, Maryann Martone.

The abstract and the introduction currently focus on describing the NFDI-Neuro project, rather than the landscape of RDM. Indeed, there is no explicit definition RDM in this context, nor a description of the scope of the survey regarding RDM. These should be found at the outset. Many of the references in the Discussion should be found earlier in the introduction. Also, some relevant references to inform many of the questions in the survey are missing, e.g.:

- Milham, M. P., Craddock, R. C., Son, J. J., Fleischmann, M., Clucas, J., Xu, H., ... Klein, A. (2018, July 19). Assessment of the impact of shared brain imaging data on the scientific literature. Nature Communications. Springer Science and Business Media LLC. http://doi.org/10.1038/s41467-018-04976-1 - providing foundations to justify why openly sharing data has pushed the field forward.

- Niso, G., Botvinik-Nezer, R., Appelhoff, S., De La Vega, A., Esteban, O., Etzel, J. A., ... Rieger, J. (2022, April 13). Open and reproducible neuroimaging: from study inception to publication. https://doi.org/10.31219/osf.io/pu5vb - Reviews the landscape of currently available tools to ensure proper RDM and transparency in neuroimaging.

- Jwa, A. S., & Poldrack, R. A. (2022, February 10). The spectrum of data sharing policies in neuroimaging data repositories. Human Brain Mapping. Wiley. http://doi.org/10.1002/hbm.25803 - Reviews existing data sharing repositories and associated reuse policies.

- Watson, C. (2022, June 21). Many researchers say they’ll share data - but don’t. Nature. Springer Science and Business Media LLC. http://doi.org/10.1038/d41586-022-01692-1 - This commentary investigates the reasons that lead scientists to state data will be made available and then fail to honor the statement.

- Longo, D. L., & Drazen, J. M. (2016, January 21). Data Sharing. New England Journal of Medicine. Massachusetts Medical Society. http://doi.org/10.1056/nejme1516564 - the infamous “research parasites” editorial in NEJM, so contrarian views are also mentioned.

- Hunt, L. T. (2019, March 4). The life-changing magic of sharing your data. Nature Human Behaviour. Springer Science and Business Media LLC. http://doi.org/10.1038/s41562-019-0560-3 - the report starts by acknowledging that most of the respondents are well-established scientists. This paper provides some reasons for ECRs to also engage in data sharing.

Another important issue is presentation. The paper includes an exhaustive list of all questions/available answers as well as large bar plots showing the responses. We agreed that going through so many separate figures generates attrition for the reader. Condensing them would enormously improve the reading and understanding.

These results need to be summarized rather than completely displayed. For instance, many of the questions (bar plots) can be summarized in a single bar that makes up for 100% of the respondents (this is only done for some of the questions, e.g., Fig. 6). In particular, Figures 1-5, 7 and 8 could be presented in a single panel. Some of the data could be presented in table format, etc. Most of the results from the survey could be condensed into a single panel. Furthermore, the specific questions should not be in the figures’ caption but in supplementary material or just described, as they are redundant with the information in the figures. Perhaps providing a copy of the survey itself as part of the downloadable files?

The current focus seems to be on the results of this particular survey, but we felt you could do a better job discussing the results in a way that could drive insights for others trying to solve these challenges. There is interesting information in the survey, but the impact gets lost in the unfocused discussion. Instead of discussing the methodological aspects of their survey or delving deeply into the results, you give a high level summary of the concerns followed by laundry list of existing efforts without commenting on why, if such existing efforts are so fabulous and well used, researchers still feel they have neither the tools, training nor expertise for RDM. You claim you will be building a Virtual Research Environment but just list the efforts without providing any insight into how it would be run or what would be expected of participants. So while the results of the survey are interesting and potentially important to the field of RDM, the rest of the manuscript does not deal with them enough. Without a deeper consideration of what the survey is trying to say regarding current and future approaches, simply presenting the results of a survey is not that interesting. It says that NFDI-Neuro will tackle all the remaining challenges in integration and data and metadata standards, but gives very short shrift to the process as to how that will be done. Many other projects have tried, some have partially succeeded, e.g., Elixir, but no mention is made of the likely challenges and what has worked elsewhere.

Although the title of the manuscript mentions the survey and overcoming the reproducibility crisis, the authors do not really engage with either topic very much. The results largely provide the results of the survey, with some brief summaries of lessons learned but little deep analysis. These summaries hint at interesting findings, e.g., that researchers who use standards are more likely to share their data, and such points should be picked up in the discussion. Instead, after laying out how NFDI-Neuro organized their priority areas based on the results, the paper barely touches on the findings. The discussion seems to be a large list of existing efforts that can be drawn upon to create a Virtual Research Environment for German Neuroscientists without deeply considering how their decisions regarding infrastructure and governance will be informed by the survey results.

The authors don’t discuss why, if so much infrastructure and so many tools are available to the German Neuroscience Community, why are they still claiming that they do not have adequate tools or training? For example, the authors specifically mention that good provenance tools have been created, yet according to Q5, only 24% of researchers use them all the time or mostly. But the discussion states that “The German neuroscience community is exemplary in its leadership in RDM and the development of provenance tools...” and at least two platforms are mentioned that have them available. Not having public repositories that host sensitive data is a concern to a lot of researchers, yet the authors say in the discussion that the EOSC VRE does exactly that. Many researchers have no knowledge of RDM methods, yet their are local resources at institutions and the authors mentioned that they have been giving on line webinars for two years on these topics. Have they had an impact? we would like to see the authors engage with the results and the topic more deeply.

Some other specific issues:

1) What about the nature of the sample, e.g., do you think that the 218 responses were representative of the community and sufficient on which to make decisions? For example, neuroimaging was very heavily represented in the responders (49%), but perhaps their needs, tools and expertise is very different from those scientists who are at the bench. Microscopic imaging/histology didn’t seem to be called out explicitly in the list of techniques (Q4). Why do 53% of researchers mostly claim that they manage their data in a way that others can use it (Q16), yet inappropriately documented code in non-reproducible environments seem to be the top or near the top concern of at least 50% of researchers (Q19)? There are interesting disconnects in the survey results compared to actual practice that are worthy of discussion.

2) There should be comparisons with other surveys that covered similar topics. For example, many researchers in surveys on data sharing cite the lack of time as a major reason why they don’t share data, and yet Q18 states that 40% of researchers claim they require less than a week. That is worthy of comment in the data sharing battles.

3) The writing in the discussion needs to be tightened up. It wasn’t always clear whether steps towards a VRE had already occurred or will occur. For example, on page 39, the authors state that NFDI will design end to end services; later in the paragraph, they say that the NFDI infrastructure is designed...” as if it exists already. Has NIFDI-Neuro already chosen DataLad and EBRAINs or are they in the process of choosing something. If so, how will that be done? How will the whole thing would work. What is the timeline? How will the community be brought into it? What percentage of the wider community use existing platforms (the section header in the discussion says “thousands”) that are available to them in Germany or, as their survey suggests, is it only certain types of researchers? It is time that we confront the usability of research infrastructures. They get created but they are often too difficulty for a non-motivated user to actually employ. Is that the case in Germany?

4) The methods say that 114 questions were asked arranged in 20 sets, but the results say they provide all survey questions. I’m not finding the 114 questions in what is presented.

5) More detail needs to be provided on the meaning of survey answers. Have these been documented somewhere? Did the respondents get more information about the meaning of the terms? Making sure that the respondents understood what the answers meant will be critical to using the results of the survey meaningfully. For example, the term “data format” is used in questions 5 and 6. I find it hard to believe that only 42% use existing tools and standards when dealing with data formats. On Q6, 66% of respondents said that “Other” was not relevant to their work. What does that mean? Some terms, e.g., neuroinformatics, and some of the responder roles, may have different meanings across different parts of the world. So definitions should be provided, or some explanation given about how these concerns were/did not need to be mitigated.

6) The main group sharing data were the RDM managers (Fig 23)? Whose data are they sharing? We assume it belongs to the researchers they are supporting. If so, then the fact that the majority of respondents seem not to have access to such staff is a problem. How will NFDI-Neuro address that?

7) EBRAINS: What does the AISBL stand for? Also, can you clarify what the difference is between EBRAINS as the coordinating unit of the HBP and EBRAINS as an infrastructure? The website seems to indicate it is the latter. What do you mean by coordinating unit? It is also somewhat misleading to say that EBRAINS started in 2013 with the HBP. I didn’t see the neuroinformatics platform referred to by that name until the last few years.

8) The respective roles of NFDI Neuro, EBRAINS and INCF: According to the introduction, “NFDI-Neuro will tackle the conceptual and logistic challenge of the integration and the development of a standardized representation of data and metadata.” As this mission does overlap with that of the other two organizations, it would be good to specify how it is different? INCF is a standards organization that supports working groups, EBRAINS is an infrastructure that has specified data and metadata standards through its knowledge graph. What will be different here?

9) Discussion: “83% of respondents did not indicate that their research data must be handled in an individual way not easily compatible with existing standards, tools, or guidelines (Fig. 15).” That is an odd phrasing. I think perhaps it should be: “Eight three percent of respondents indicated that their research data did not have to be handled in an individual way...” or Seventeen percent of respondents indicated that their data must be handled in an individual way...”

10) Title: The title makes it seem as if the survey and study will be about reproducibility, but it is primarily about research data management and infrastructure. Both are integral to reproducibility but are only part of the problem. The opening sentence of the introduction introduces the reproducibility crisis, but then goes on to describe RDM. It then describes research infrastructure and its intended use for artificial intelligence and computational modeling without tying either of these to reproducibility. In fact the study is more about the latter two than the former. The survey does touch on some issues involved in reproducible science but it was mostly self assessment. Therefore, the title should be changed to be more appropriate for the scope of the paper.

11) Has anything been learned from other large infrastructure/data sharing projects in Europe that will help this effort succeed? e.g., Elixir or the HBP?

## Author Response

General comment.

We thank the reviewers for their time and effort in providing this extensive, detailed and very constructive review. We have addressed all concerns of the reviewers in the revised version of the manuscript.

Reviewer:

Computational Neuroscience Model Code Accessibility Comments for Author (Required):

A data availability statement is missing. Providing the data with a working link to some sharing site such as github is necessary, and more important than describing the license (typically the license information would be provided at the download site, not in the paper itself). Similarly, the manuscript mentions R scripts to generate the figures, and this code should be available at the same site. We also recommend the survey data be placed there, which will greatly debulk the paper itself (see below).

Answer:

All data including the original survey data and R scripts are publicly available. We have now placed the link to the data more prominently in the manuscript as follows:

“The survey and related collected data, as well as all analysis scripts are available publicly (https://doi.org/10.12751/g-node.w5h68v).”

We have also removed the license information from the manuscript as requested.

Reviewer:

The abstract and the introduction currently focus on describing the NFDI-Neuro project, rather than the landscape of RDM. Indeed, there is no explicit definition RDM in this context, nor a description of the scope of the survey regarding RDM. These should be found at the outset. Many of the references in the Discussion should be found earlier in the introduction. Also, some relevant references to inform many of the questions in the survey are missing, e.g.:

- Milham, M. P., Craddock, R. C., Son, J. J., Fleischmann, M., Clucas, J., Xu, H., ... Klein, A. (2018, July 19). Assessment of the impact of shared brain imaging data on the scientific literature. Nature Communications. Springer Science and Business Media LLC. http://doi.org/10.1038/s41467-018-04976-1 - providing foundations to justify why openly sharing data has pushed the field forward.

- Niso, G., Botvinik-Nezer, R., Appelhoff, S., De La Vega, A., Esteban, O., Etzel, J. A., ... Rieger, J. (2022, April 13). Open and reproducible neuroimaging: from study inception to publication. https://doi.org/10.31219/osf.io/pu5vb - Reviews the landscape of currently available tools to ensure proper RDM and transparency in neuroimaging.

- Jwa, A. S., & Poldrack, R. A. (2022, February 10). The spectrum of data sharing policies in neuroimaging data repositories. Human Brain Mapping. Wiley. http://doi.org/10.1002/hbm.25803 - Reviews existing data sharing repositories and associated reuse policies.

- Watson, C. (2022, June 21). Many researchers say they’ll share data - but don’t. Nature. Springer Science and Business Media LLC. http://doi.org/10.1038/d41586-022-01692-1 - This commentary investigates the reasons that lead scientists to state data will be made available and then fail to honor the statement.

- Longo, D. L., & Drazen, J. M. (2016, January 21). Data Sharing. New England Journal of Medicine. Massachusetts Medical Society. http://doi.org/10.1056/nejme1516564 - the infamous “research parasites” editorial in NEJM, so contrarian views are also mentioned.

- Hunt, L. T. (2019, March 4). The life-changing magic of sharing your data. Nature Human Behaviour. Springer Science and Business Media LLC. http://doi.org/10.1038/s41562-019-0560-3 - the report starts by acknowledging that most of the respondents are well-established scientists. This paper provides some reasons for ECRs to also engage in data sharing.

Answer:

We thank the reviewer for this comment and for pointing to the additional relevant literature.

As suggested, we have revised the abstract to focus more on the RDM landscape. We also incorporated the suggested references and cite multiple relevant references of the discussion now earlier in the introduction. As requested, now the abstract gives a description of the scope of the survey regarding RDM as follows:

“To obtain an understanding of the present RDM situation in the neuroscience community, NFDI-Neuro conducted a comprehensive survey amongst the neuroscience community. Here, we report and analyse the results of the survey. We focused the survey and our analysis on current needs, challenges, and opinions about RDM to use this information to shape the specific work plan for the NFDI-Neuro initiative.”

We now also provide a definition of RDM as requested:

“RDM describes the organization, storage, preservation, and sharing of scientific data. A particular focus is on the day-to-day management of research data during the lifetime of a research project and on the long-term usability of these data according to FAIR principles (findable, accessible, interoperable and reusable; Wilkinson et al., 2016).”

Reviewer:

Another important issue is presentation. The paper includes an exhaustive list of all questions/available answers as well as large bar plots showing the responses. We agreed that going through so many separate figures generates attrition for the reader. Condensing them would enormously improve the reading and understanding.

These results need to be summarized rather than completely displayed. For instance, many of the questions (bar plots) can be summarized in a single bar that makes up for 100% of the respondents (this is only done for some of the questions, e.g., Fig. 6). In particular, Figures 1-5, 7 and 8 could be presented in a single panel. Some of the data could be presented in table format, etc. Most of the results from the survey could be condensed into a single panel. Furthermore, the specific questions should not be in the figures’ caption but in supplementary material or just described, as they are redundant with the information in the figures. Perhaps providing a copy of the survey itself as part of the downloadable files?

Answer:

As suggested, we have abandoned the idea of presenting all questions graphically in the paper.

We have moved the visual representation of the questions to the supplementary materials and now focus on the most relevant results in the Results section, which we present now in a more condensed form.

Reviewer:

The current focus seems to be on the results of this particular survey, but we felt you could do a better job discussing the results in a way that could drive insights for others trying to solve these challenges. There is interesting information in the survey, but the impact gets lost in the unfocused discussion. Instead of discussing the methodological aspects of their survey or delving deeply into the results, you give a high level summary of the concerns followed by laundry list of existing efforts without commenting on why, if such existing efforts are so fabulous and well used, researchers still feel they have neither the tools, training nor expertise for RDM. You claim you will be building a Virtual Research Environment but just list the efforts without providing any insight into how it would be run or what would be expected of participants. So while the results of the survey are interesting and potentially important to the field of RDM, the rest of the manuscript does not deal with them enough. Without a deeper consideration of what the survey is trying to say regarding current and future approaches, simply presenting the results of a survey is not that interesting. It says that NFDI-Neuro will tackle all the remaining challenges in integration and data and metadata standards, but gives very short shrift to the process as to how that will be done. Many other projects have tried, some have partially succeeded, e.g., Elixir, but no mention is made of the likely challenges and what has worked elsewhere.

Answer:

To address these reviewer comments, we rewrote the discussion in large parts, and we now focus more on the results in a way that could drive insights for others trying to solve these challenges.

For example: we have deleted the “Roadmap: Establish a federated interoperable ecosystem for data and reproducible research” (“laundry list”) and condensed the discussion significantly including the discussion of the methodological aspects of the survey. This gave us room for a more detailed discussion of the results especially the existing barriers and the insufficient incentives to spend the time needed for RDM. We also discuss the need for training in the context of the survey instead of the NFDI proposal.

Reviewer:

Although the title of the manuscript mentions the survey and overcoming the reproducibility crisis, the authors do not really engage with either topic very much. The results largely provide the results of the survey, with some brief summaries of lessons learned but little deep analysis. These summaries hint at interesting findings, e.g., that researchers who use standards are more likely to share their data, and such points should be picked up in the discussion. Instead, after laying out how NFDI-Neuro organized their priority areas based on the results, the paper barely touches on the findings. The discussion seems to be a large list of existing efforts that can be drawn upon to create a Virtual Research Environment for German Neuroscientists without deeply considering how their decisions regarding infrastructure and governance will be informed by the survey results.

Answer:

We agree with the reviewer and have changed the title of the manuscript to: “Research Data Management and Data Sharing for Reproducible Research - Results of a Community Survey of the German National Research Data Infrastructure Initiative Neuroscience.”

We have also revised the discussion accordingly.

Reviewer:

The authors don’t discuss why, if so much infrastructure and so many tools are available to the German Neuroscience Community, why are they still claiming that they do not have adequate tools or training? For example, the authors specifically mention that good provenance tools have been created, yet according to Q5, only 24% of researchers use them all the time or mostly. But the discussion states that “The German neuroscience community is exemplary in its leadership in RDM and the development of provenance tools...” and at least two platforms are mentioned that have them available. Not having public repositories that host sensitive data is a concern to a lot of researchers, yet the authors say in the discussion that the EOSC VRE does exactly that. Many researchers have no knowledge of RDM methods, yet their are local resources at institutions and the authors mentioned that they have been giving on line webinars for two years on these topics. Have they had an impact? we would like to see the authors engage with the results and the topic more deeply.

Answer:

We agree with the concern of the Reviewer. We rewrote the discussion in large parts and the discussion has now a stronger focus on the results of our survey and insights drawn from these.

For example: The Section 4 where the reviewer cited “The German neuroscience community is exemplary in its leadership in RDM and the development of provenance tools, s” were completely removed as we agree that this subsection give the reader no additional insights into how to solve the existing problems and is not associated with the survey enough. We have also deleted the roadmap to “Establish a federated interoperable ecosystem for data and reproducible research” as this again gives the reader not much additional insight into the results of the survey.

This gave us room for a more detailed discussion of the results especially the existing barriers and the insufficient incentives to spend the time needed for RDM.

Reviewer:

Some other specific issues:

1) What about the nature of the sample, e.g., do you think that the 218 responses were representative of the community and sufficient on which to make decisions? For example, neuroimaging was very heavily represented in the responders (49%), but perhaps their needs, tools and expertise is very different from those scientists who are at the bench. Microscopic imaging/histology didn’t seem to be called out explicitly in the list of techniques (Q4). Why do 53% of researchers mostly claim that they manage their data in a way that others can use it (Q16), yet inappropriately documented code in non-reproducible environments seem to be the top or near the top concern of at least 50% of researchers (Q19)? There are interesting disconnects in the survey results compared to actual practice that are worthy of discussion.

Answer:

We agree with the Reviewer. We have added the topic of representativeness to the discussion as follows:

“This result is consistent with a previous survey on open science practices in functional neuroimaging which reported that 34% of their participants have never shared their raw neuroimaging data (Paret et al., 2021). This similarity could also be caused by the fact that 49% or respondents in our survey are engaged in neuroimaging. Some other branches might be underrepresented like Cellular Molecular Neuroscience (11%) or Neuroinformatics (14%). The sample size of the survey is comparable to other surveys in this area and similarity of results indicate representativeness (Niso et al., 2022).”

With respect to the finding that 53% of researchers mostly claim that they manage their data in a way that others can use it (Q16), yet inappropriately documented code in non-reproducible environments seem to be the top or near the top concern of at least 50% of researchers (Q19),

we speculate about following possible explanations: If 47% of scientists do not manage their data in a way that others can use, this would explain the problem of insufficiently documented code. Another possibility would be that scientists do try to use the tools available and do use them in 9 out of 10 steps of data analysis and preprocessing. However, if they want to add another new experimental analysis step in a single step, then they may be creating inadequately documented code in non-reproducible environments.

Reviewer:

2) There should be comparisons with other surveys that covered similar topics. For example, many researchers in surveys on data sharing cite the lack of time as a major reason why they don’t share data, and yet Q18 states that 40% of researchers claim they require less than a week. That is worthy of comment in the data sharing battles.

Answer:

We agree with the Reviewer and now compare our results to other surveys (Paret et al., 2021).

We discuss the time demand now in more detail as follows:

“The barriers to share data applies also to the requirement of data preparation. This is a time-consuming process. 70% of those respondents who had previously prepared data for publication and re-use indicated that the time they need to ready a dataset requires more than a day, and 26% need even more than a week (Fig. 6). Accordingly, 60% thought that there is lack of time to deposit data in a repository. In comparison, only 23% did not believe that time is a problem for depositing data in a public repository.

In other words, 40% of scientists say it takes them less than a week to prepare a dataset for publication and reuse. The fact that 60% nevertheless think there is a lack of time suggests that scientists do not think the time investment is worth it.

The lack of time results from competing demands, each of which requires time. Given that scientists do science, this result suggests that preparing data for publication and reuse competes with the act of doing science, which itself is not always perceived as a central part of science. In any case, however, the result of a lack of time indicates that scientists perceive other tasks as more essential. Accordingly, this is a reflection of an insufficient incentive to spend the time to prepare data for publication and reuse.”

Reviewer:

3) The writing in the discussion needs to be tightened up. It wasn’t always clear whether steps towards a VRE had already occurred or will occur. For example, on page 39, the authors state that NFDI will design end to end services; later in the paragraph, they say that the NFDI infrastructure is designed...” as if it exists already. Has NIFDI-Neuro already chosen DataLad and EBRAINs or are they in the process of choosing something. If so, how will that be done? How will the whole thing would work. What is the timeline? How will the community be brought into it? What percentage of the wider community use existing platforms (the section header in the discussion says “thousands”) that are available to them in Germany or, as their survey suggests, is it only certain types of researchers? It is time that we confront the usability of research infrastructures. They get created but they are often too difficulty for a non-motivated user to actually employ. Is that the case in Germany?

Answer:

We agree with the Reviewer and rewrote the discussion. With respect to the overall direction of the reviewer comments we now have a stronger focus on the discussion of the survey results and less on the work program of NFDI.

Reviewer:

4) The methods say that 114 questions were asked arranged in 20 sets, but the results say they provide all survey questions. I’m not finding the 114 questions in what is presented.

Answer:

We agree with the Reviewer that this statement might be confusing. We have revised the description as follows to clarify the issue:

“The NFDI-Neuro survey comprised 20 sets of questions, where each set contains one or multiple questions. Counting all questions yields a total of 114 questions presented to each survey participant.”

Reviewer:

5) More detail needs to be provided on the meaning of survey answers. Have these been documented somewhere? Did the respondents get more information about the meaning of the terms? Making sure that the respondents understood what the answers meant will be critical to using the results of the survey meaningfully. For example, the term “data format” is used in questions 5 and 6. I find it hard to believe that only 42% use existing tools and standards when dealing with data formats. On Q6, 66% of respondents said that “Other” was not relevant to their work. What does that mean? Some terms, e.g., neuroinformatics, and some of the responder roles, may have different meanings across different parts of the world. So definitions should be provided, or some explanation given about how these concerns were/did not need to be mitigated.

Answer:

Participants in this survey were not given any further information about the meaning of specific terms. The survey was answered as the participants understood the terms.

From a practical standpoint, it would be difficult to ensure a common understanding of all terms because (as the reviewer noted) it would be necessary to ensure that a particular definition was used by respondents. Since it is already difficult to motivate subjects for such surveys, the proposed steps would create another hurdle although we agree that it would improve the interpretability of the results. As the reviewer requested, we have added this concern in the limitation section as follows:

“Participants in this survey were not given any further information about the meaning of each term. The survey was answered as the participants understood the terms. Different levels of knowledge and also different sub-disciplines could have an influence on the understanding of the terminology which in turn could affect the interpretation of the results.”

Reviewer:

6) The main group sharing data were the RDM managers (Fig 23)? Whose data are they sharing? We assume it belongs to the researchers they are supporting. If so, then the fact that the majority of respondents seem not to have access to such staff is a problem. How will NFDI-Neuro address that?

Answer:

The question of whose data the RDM managers sharing was not ask in the survey. We can also only speculate about the answer.

Reviewer:

7) EBRAINS: What does the AISBL stand for? Also, can you clarify what the difference is between EBRAINS as the coordinating unit of the HBP and EBRAINS as an infrastructure? The website seems to indicate it is the latter. What do you mean by coordinating unit? It is also somewhat misleading to say that EBRAINS started in 2013 with the HBP. I didn’t see the neuroinformatics platform referred to by that name until the last few years.

Answer:

EBRAINS is an AISBL (Association Internationale Sans But Lucratif) under Belgian Law which means that it is an international association in a non-profit legal form.

However, in regard to this and the other questions and according to the other reviewer comments, we have rewritten the discussion to large parts and have deleted the section “Roadmap: Establish a federated interoperable ecosystem for data and reproducible research” in which EBRAINS, HBP and AISBL was discussed. The terms “HBP” and “AISBL” now no longer appear in the manuscript.

Reviewer:

8) The respective roles of NFDI Neuro, EBRAINS and INCF: According to the introduction, “NFDI-Neuro will tackle the conceptual and logistic challenge of the integration and the development of a standardized representation of data and metadata.” As this mission does overlap with that of the other two organizations, it would be good to specify how it is different? INCF is a standards organization that supports working groups, EBRAINS is an infrastructure that has specified data and metadata standards through its knowledge graph. What will be different here?

Answer:

With respect to the other comments of the reviewer, we focus the manuscript on the survey and the results and less on NFDI, EBRAINS and INCF. Therefore, we refrain from discussing this topic.

Reviewer:

9) Discussion: “83% of respondents did not indicate that their research data must be handled in an individual way not easily compatible with existing standards, tools, or guidelines (Fig. 15).” That is an odd phrasing. I think perhaps it should be: “Eight three percent of respondents indicated that their research data did not have to be handled in an individual way...” or Seventeen percent of respondents indicated that their data must be handled in an individual way...”

Answer:

We agree with the Reviewer and have changed the sentence to:

“Eighty-three percent of respondents indicated that their research data did not have to be handled in an individual way not easily compatible with existing standards, tools, or guidelines (Fig. 4).”

Reviewer:

10) Title: The title makes it seem as if the survey and study will be about reproducibility, but it is primarily about research data management and infrastructure. Both are integral to reproducibility but are only part of the problem. The opening sentence of the introduction introduces the reproducibility crisis, but then goes on to describe RDM. It then describes research infrastructure and its intended use for artificial intelligence and computational modeling without tying either of these to reproducibility. In fact the study is more about the latter two than the former. The survey does touch on some issues involved in reproducible science but it was mostly self assessment. Therefore, the title should be changed to be more appropriate for the scope of the paper.

Answer:

We agree with the reviewer and have changed the title. With respect to this and the other comments, we have extensively revised the manuscript. The focus of the manuscript is now on the survey results and the discussion of these results with respect to RDM.

Reviewer:

11) Has anything been learned from other large infrastructure/data sharing projects in Europe that will help this effort succeed? e.g., Elixir or the HBP?

Answer:

In respect to the overall direction of reviewer comments we now have a stronger focus on the discussion of the survey results and less on the work program of NFDI or other large infrastructure/data sharing projects in Europe. We, therefore, think that a discussion of this very complex question is beyond the scope of the current manuscript.
